# Differential influences of environment and self-motion on place and grid cell firing

**DOI:** 10.1038/s41467-019-08550-1

**Published:** 2019-02-07

**Authors:** Guifen Chen, Yi Lu, John A King, Francesca Cacucci, Neil Burgess

**Affiliations:** 1 UCL Institute of Cognitive Neuroscience, University College London, 17 Queen Square, London, WC1N 3AZ UK; 2 Department of Neuroscience Physiology & Pharmacology, University College London, Gower Street, London, WC1E 6BT UK; 3 Department of Clinical Educational Health Psychology, University College London, Gower Street, London, WC1E 6BT UK; 4 UCL Queen Square Institute of Neurology, University College London, Queen Square, London, WC1N 3BG UK

## Abstract

Place and grid cells in the hippocampal formation provide foundational representations of environmental location, and potentially of locations within conceptual spaces. Some accounts predict that environmental sensory information and self-motion are encoded in complementary representations, while other models suggest that both features combine to produce a single coherent representation. Here, we use virtual reality to dissociate visual environmental from physical motion inputs, while recording place and grid cells in mice navigating virtual open arenas. Place cell firing patterns predominantly reflect visual inputs, while grid cell activity reflects a greater influence of physical motion. Thus, even when recorded simultaneously, place and grid cell firing patterns differentially reflect environmental information (or ‘states’) and physical self-motion (or ‘transitions’), and need not be mutually coherent.

## Abstract (web summary)

Place cells and grid cells are known to encode spatial information about an animal’s location relative to the surrounding environment. Here, the authors show that place cells predominantly encode environmental sensory inputs, while grid cell activity reflects a greater influence of physical motion.

## Introduction

The spatial firing patterns of place cells and grid cells provide a window into how we represent environmental location^1,2^ and potentially how we organise conceptual knowledge^3,4^. However, it is not clear how these spatial representations are formed. Place and grid cells might represent different sources of spatial information provided by the sensory environment and by self-motion^5–7^, or they might form a single coherent representation in which either place or grid cell firing is strongly influenced by the other cell type^8–10^. The unitary firing fields of place cells, their tendency to ‘remap’ between environments with different sensory attributes^11^ and to change parametrically following environmental changes^12^ indicate a strong influence of environmental information on place cell firing. By contrast, the regular periodic firing patterns of grid cells, maintained across different environments, indicate a strong intrinsic organisation thought to be driven by self-motion inputs^2,5–7^. However, place cell firing patterns are influenced by self-motion^13^, and grid cell firing patterns by environmental sensory inputs^2,14–16^. Crucially, the relative influence of self-motion and environmental sensory inputs on the firing of place and grid cells within a given animal has not been quantified, and we do not know whether the two cell types integrate these inputs separately, or combine them to provide a single holistic representation.

Normally, self-motion drives corresponding changes in environmental inputs, so the two cannot be dissociated. However, virtual reality (VR) can be used to manipulate the relationship between physical (motoric/proprioceptive) self-motion signals and environmental visual information (including both identifiable landmarks and optic flow) so that their relative influences can be identified. This approach has been used on 1-dimentional (1-d) virtual tracks while recording from place cells^17^ or grid cells^18^, suggesting that both types of input can influence the pattern of firing along the track in both types of cells, in ways that vary across cells^17^ and conditions^18^, see Discussion.

Here we decoupled the physical self-motion and environmental visual signals available to mice running in 2-d virtual open field environments, while recording from place and grid cells. We then compared the relative influences of these two types of information on the scales of the characteristic 2-d spatial firing patterns of place and grid cells. We used a VR system for mice, following a similar system for rats^19,20^, which allows navigation and expression of spatial firing patterns within 2-d open field virtual environments^21^.

Within the VR system, the effects of running on a Styrofoam ball are used to drive movement of the viewpoint of the visual projection of the environment. In the baseline configuration, movement of 1 unit of distance on the surface of the ball is translated to 1 unit of movement of the viewpoint within the virtual environment: the ‘gain’ *G* between vision and movement is 1. Changes to this ‘gain’ allow differences between the distance indicated by the visual movement of viewpoint and the physical movement of the body. Under ‘increased’ gain ratios (*G* > 1), the visual projection moves further than the surface of the ball, with the opposite for decreased gain ratios (*G* < 1).

The visual appearance of the environment (e.g. aspect ratio) was kept constant across trials, with changes to gain affecting the physical distance run across the environment rather than its visual extent. The visual gain was changed for movement along one dimension only (the *x* axis), so that the remaining (unchanged) dimension provides a within-trial control for comparison and to identify any potentially confounding (non-spatial) effects, such as surprise or uncertainty. Finally, the use of VR removes potentially confounding local cues to location, whilst slightly reducing the overall strength of spatial coding^21^.

In summary, place cell firing patterns predominantly reflect visual inputs, while grid patterns reflect a much greater influence of physical motion. Thus, even when recorded simultaneously, place and grid cell firing patterns differentially reflect environmental information and physical self-motion, and need not be mutually coherent.

## Results

### The ‘gain’ of the mapping from physical to visual motion

**Fig. 1:**
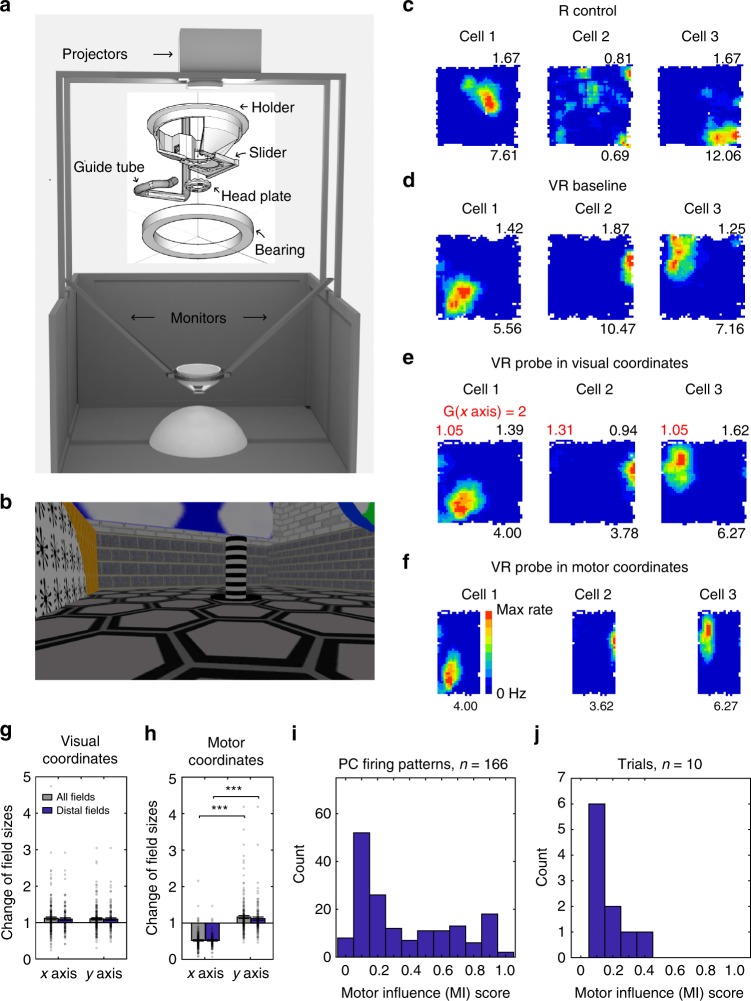
Place cell firing patterns under visual gain increase. **a**, **b** Schematic of the VR setup (**a**, inset: the rotating head-holder) and view from inside the VR environment, the striped column indicates a visible reward location (**b**, see Supplementary Video). Adapted from ref. ^21^. **c**–**f** Three place cells simultaneously recorded in a 60 × 60 cm square box (**c**), a 60 × 60 cm virtual square environment (**d**), and a probe trial where visual gain was increased along the *x* axis (*G* = 2) plotted in visual coordinates (**e**) and motor coordinates (**f**). Firing rate maps shown with max rate below (Hz) and spatial information above (bits/spike), stretch factor *F* mapping visual plots to baseline shown in red, all three trials recorded on the same day. **g**, **h** Change of place field size (ratio relative to baseline) was significantly larger on the manipulated than un-manipulated axis when plotted in motor coordinates (**h**, *n* = 173 for all fields, paired *t*-test, *t*(172) = −14.35, *p* < 0.001; *n* = 112 for fields distal to boundaries, *t*(111) = −12.22, *p* < 0.001) but not when plotted in visual coordinates (**g**; *n* = 173 for all fields, *t*(172) = 0.29, *p* = 0.77; *n* = 112 for distal fields, *t*(111) = −0.03, *p* = 0.98), reflecting strong visual influence. **i**, **j** Distribution of motor influence scores ‘MI’ based on firing rate maps (**i**) or on population vectors (**j**). Note MI = (*F*−1)/(*G*−1), so MI = 0 indicates firing in visual coordinates (i.e. *F* = 1 implies no effect of *G* ≠ 1); MI = 1 indicates motor coordinates (i.e. *F* = *G*: visual stretch factor equals visual gain factor)

We examined the spatial firing patterns of place cells from hippocampal region CA1 and grid cells from medial Entorhinal cortex (mEC) in 2-d VR, focussing on probe trials in which the visual ‘gain’ (*G*) applied to one axis of virtual movement was both increased (*G* = 2) and decreased (*G* = 2/3) compared to the baseline condition (*G* = 1). An experimental day comprised a baseline (*G* = 1) VR trial, a probe (*G*≠1) trial and a real-world trial. Under non-unity gain, firing rates can be plotted against the animal’s location according to vision (in visual coordinates) or physical movement (in motor coordinates). These plots are identical for baseline trials. See Fig. 1 and Methods for details and Supplementary Figures 1–2 for examples of simultaneously recorded place and grid cell firing patterns. Overall, we found that there were no significant differences in mean firing rates and spatial information between the baseline and probe conditions, although decreases in peak firing rates and gridness scores were observed in the probe conditions (Supplementary Figure 3).

### Place cell firing patterns

**Fig. 2:**
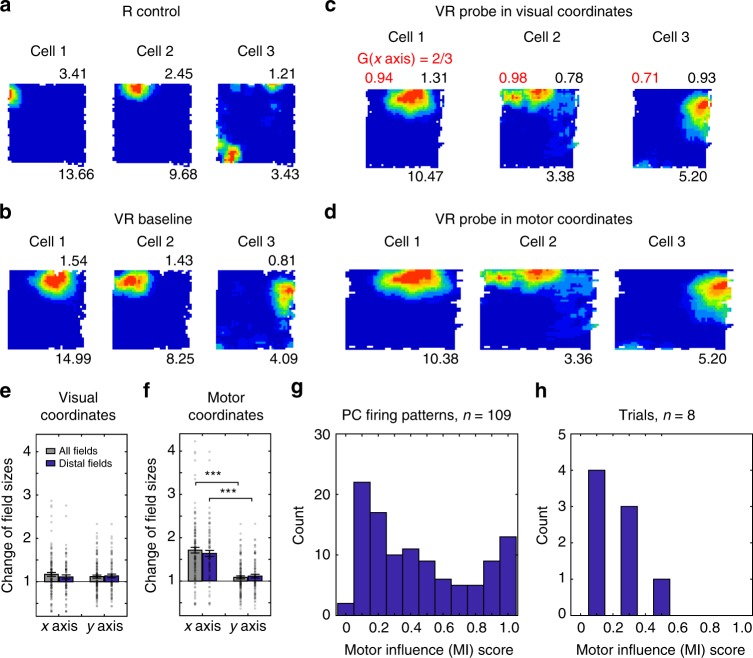
Place cell firing patterns under visual gain decrease. **a**, **b** Three place cells simultaneously recorded in a 60 × 60 cm square box (**a**), a 60 × 60 cm virtual square (**b**), and a probe trial where visual gain was reduced along the *x* axis (*G* = 2/3) plotted in motor coordinates (**c**) and visual coordinates (**d**). Layout as Fig. 1c–f, stretch factor *F* mapping visual plots to baseline shown in red. **e**, **f** Change of place field size was significantly larger on the manipulated than un-manipulated axis when plottedi in motor coordinates (**f**; *n* = 122 for all fields, paired *t*-test, *t*(121) = 9.77, *p* < 0.001; *n* = 84 for fields distal to boundaries, *t*(83) = 7.71, *p* < 0.001) but not when plotted in visual coordinates (**e**; *n* = 122 for all fields, *t*(121) = 1.07, *p* = 0.29; *n* = 84 for distal fields, *t*(83) = −0.45, *p* = 0.65), reflecting strong visual influence. (**g**, **h**) Distribution of motor influence scores ‘MI’ based on firing rate maps (**g**) or population vectors (**h**; see Methods and Fig. 1)

We first compared place cell firing patterns between probe and baseline trials. Figures 1 and 2 show firing patterns for place cells plotted against visual or motor location under increases (*G* = 2) and decreases (*G* = 2/3) in visual gain, respectively. Firing fields had similar sizes along the manipulated dimension in probe trials compared to baseline trials when plotted in visual coordinates (Figs. 1g and 2e), but different sizes when plotted in motor coordinates (Figs. 1h and 2f), indicating predominantly visual coding. Field sizes along the un-manipulated dimension did not change between probe and baseline trials (paired *t*-tests, *t*(111) = 1.04, *p* = 0.30 for gain increase; *t*(83) = 1.55, *p* = 0.13 for gain decrease).

We can quantify the relative influence of physical motion versus visual input on a cell’s spatial firing pattern (i.e. the firing rate map, plotted in visual coordinates) by comparing it to a stretched version of the baseline firing rate map, and finding the stretch factor (*F*) giving the best fit (allowing for all offsets of the smaller to the larger map; see Methods). For probe trials (i.e., *G* ≠ 1), the ‘motor influence’ score MI = (*F*−1)/(*G*−1) varies from 0 for firing patterns that resemble baseline patterns (i.e. *F* = 1; no effect of having to run more or less than expected from vision), to 1 for firing patterns that show stretching corresponding to the gain manipulation (i.e. *F* = *G*; the pattern in visual coordinates appears to stretch according to the gain relating vision to movement).

Place cell firing patterns can ‘remap’^11^ or become diffuse, making comparisons difficult (e.g. if a cell fires over most of the arena, the field shape largely reflects the shape of the arena). Accordingly, we confined our analyses to compact firing patterns that did not remap between baseline and probe (occupying < 50% of the arena and having a spatial correlation > 0.3 between baseline and the best fitting probe firing rate map, i.e. taking account of any gain-related offset and stretch), leaving 275/497 firing patterns (166/282 for gain increase, 109/215 for gain decrease, see Methods and Supplementary Figure 4). The percentage of remapping varied across sessions (median: 12%, interquartile range: 16%) but did not differ between gain increase and decrease sessions (Supplementary Figure 5). The results from both types of gain manipulation show an overall greater visual than motor influence on place cell firing (median = 0.21, interquartile range (IQR) = 0.53 for MI scores in the gain increase, and median = 0.37, IQR = 0.59 in the gain decrease, see Figs. 1i and 2g and S6).

We also analysed the population vector responses of all simultaneously recorded place cells, finding the best single offset and stretch factor for each population (in 18 trials with multiple place and grid cells), again finding greater visual than motor influence on place cell populations (MI score in gain increase: median = 0.08, IQR = 0.11; in gain decrease: median = 0.21, IQR = 0.21, see Figs. 1j and 2h and S7).

### Grid cell firing patterns

**Fig. 3:**
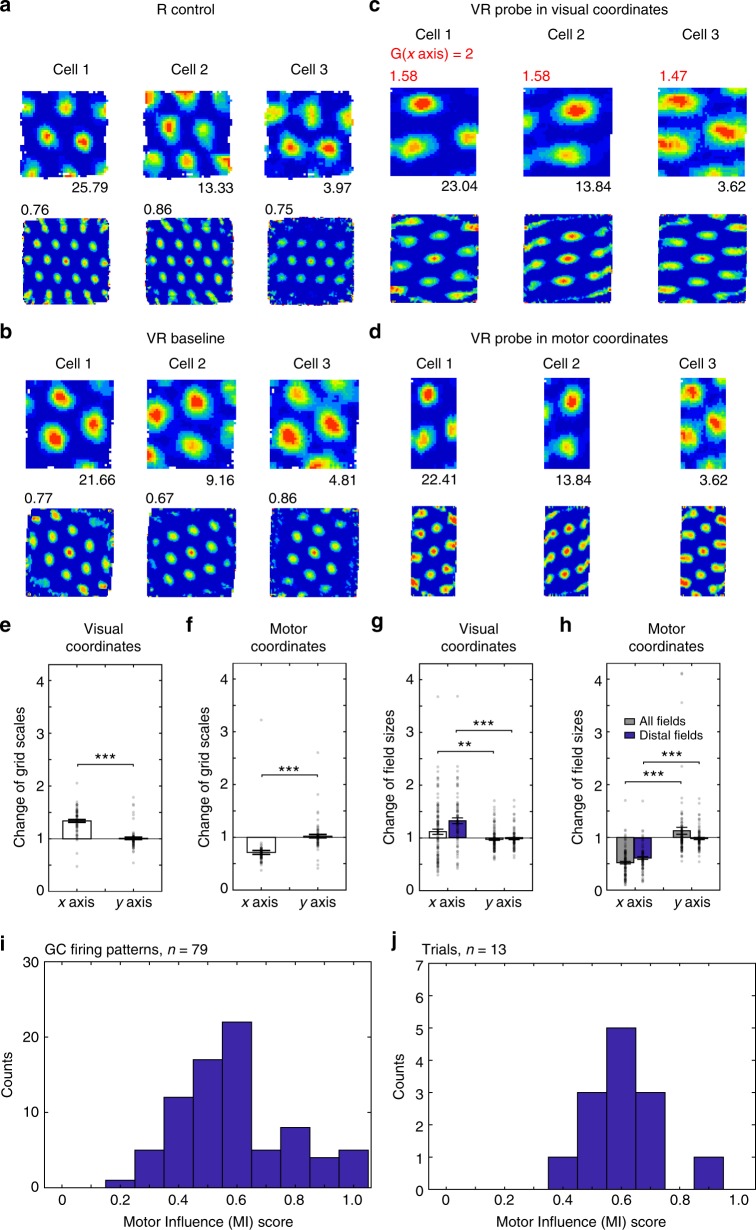
Grid cell firing patterns under visual gain increase. **a**–**d** Three grid cells simultaneously recorded in the baseline 60 × 60 cm square box (**a**), a 60 × 60 cm virtual square environment (**b**) and a virtual probe trial where the gain of visual motion compared to physical motion on the ball was increased along the *x* axis (gain *G* = 2), plotted in visual coordinates (**c**) and motor coordinates (**d**). Firing rate maps (and max rates) shown above, spatial autocorrelograms (and gridness scores) below, one cell per column, stretch factor *F* mapping visual plots to baseline shown in red, all three trials recorded on the same day. **e**, **f** Change of grid scales was significantly larger on the manipulated than un-manipulated axis when plotted in visual coordinates (**e**; *n* = 72, paired *t*-test, *t*(71) = 9.68, *p* < 0.001) or motor coordinates (**f**; *n* = 72, *t*(71) = −5.61, *p* < 0.001). **g**, **h** Change of firing field size was significantly larger on the manipulated than un-manipulated axis when plotted in both visual coordinates (**g**; *n* = 117 for all fields, *t*(116) = 2.90, *p* < 0.01; *n* = 83 for distal fields, *t*(82) = 6.27, *p* < 0.001) and in motor coordinates (**h**; *n* = 117 for all fields, *t*(116) = −8.09, *p* < 0.001; *n* = 83 for fields distal to boundaries, *t*(82) = −12.14, *p* < 0.001). **i**, **j** Distribution of motor influence scores ‘MI’ based on firing rate maps (**i**) or population vectors (**j**; see Methods or Fig. 1)

Figure 3 shows firing patterns for grid cells plotted against visual or motor location under increases in visual gain (*G* = 2). Again we restricted analyses to grid patterns that remained similar between baseline and probe (i.e. showing spatial correlation > 0.3 after accounting for gain-related offsets or stretches), giving 79/80 cells in gain increase days and 39/42 in gain decrease days. Firing patterns appear elliptical in probe trials when plotted in visual coordinates (increased in scale along the manipulated axis, as movement in visual space requires less physical movement) and in motor coordinates (decreased in scale, as physical movement produces greater visual movement), as shown by the changes in grid scale (Fig. 3e, f). Equally the motor influence scores for firing rate maps and population vectors (median = 0.58, IQR = 0.21, Fig. 3i, and median = 0.63, IQR = 0.25, Fig. 3j) indicate a balance between motor and visual influence, weighted towards motor.

We also analysed the shapes of pairs of individual firing fields that remained similar between probe and baseline trials (showing spatial correlation > 0.3 after accounting for gain-related offsets and stretches and setting the rest of the map to zero), and that were sufficiently centrally placed to avoid occlusion by the edge of the environment (115/167 grid fields). The changes in sizes of individual fields showed a similar pattern of both visual and motor influence to the overall grid patterns (see Fig. 3g, h, and Methods for details).

**Fig. 4:**
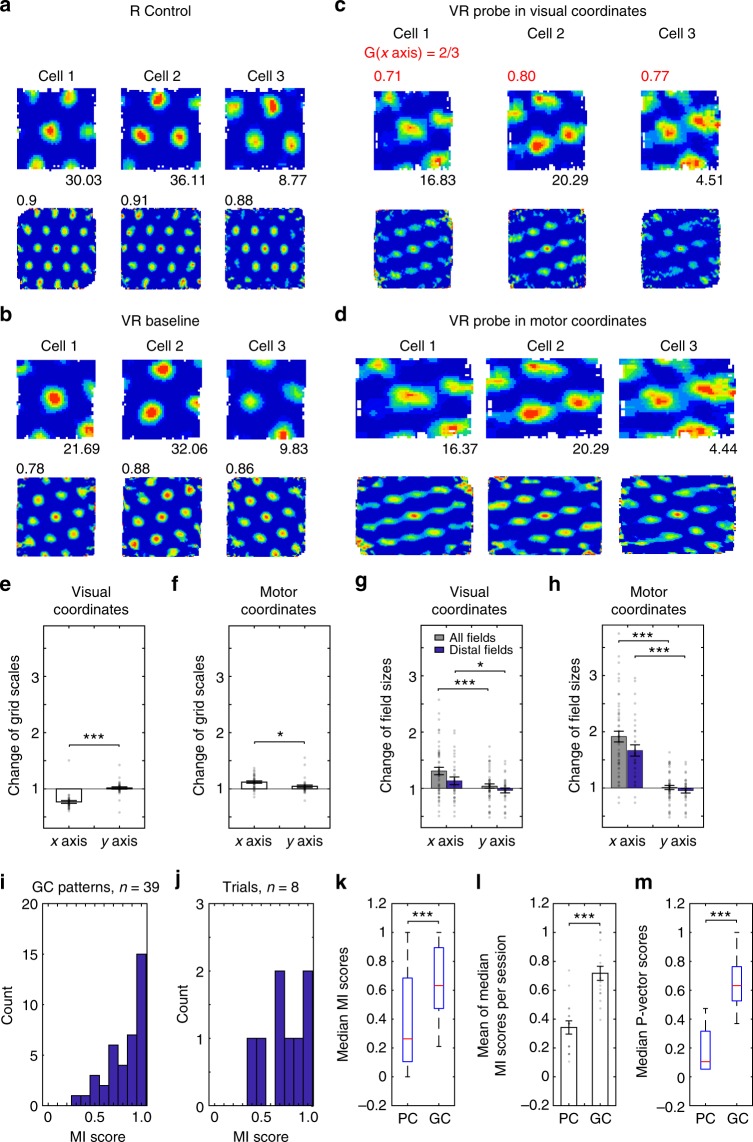
Grid cell firing patterns under visual gain decrease. **a**–**d** Three grid cells simultaneously recorded in a 60 × 60 cm square box (**a**), a 60 × 60 cm virtual square (**b**) and a probe trial where visual gain was reduced along the *x* axis (*G* = 2/3) plotted in visual coordinates (**c**) and motor coordinates (**d**), on the same day, layout as Fig. 3a–d, stretch factor *F* mapping visual plots to baseline shown in red. **e**, **f** Change of grid scales was significantly larger on the manipulated than un-manipulated axis when plotted in visual coordinates (**e**; *n* = 35, paired *t*-test, *t*(34) = -6.31, *p* < 0.001), with a smaller effect when plotted in motor coordinates (**f**; *n* = 35, *t*(34) = 3.00, *p* < 0.05). **g**, **h** Change of firing field size was significantly larger on the manipulated than un-manipulated axis when plotted in visual coordinates (**g**; *n* = 50 for all fields, *t*(49) = 3.96, *p* < 0.001; *n* = 32 for distal fields, *t*(31) = 2.11, *p* < 0.05) and even more strongly when plotted in motor coordinates (**h**; *n* = 50 for all fields, *t*(49) = 9.49, *p* < 0.001; *n* = 32 for distal fields, *t*(31) = 6.42, *p* < 0.001). **i**, **j** Distribution of motor influence score (MI) based on firing rate maps (**i**) or population vectors (**j**; see Methods or Fig. 1). **k** Median MI scores of non-remapped place and grid cell firing patterns (median (red line) = 0.26, interquartile range (IQR, *q*_1_−*q*_3_, blue box) = 0.58 for place cells, median = 0.63, IQR = 0.42 for grid cells, Wilcoxon rank sum test, *z* = −7.83, *p* < 0.001). **l** Mean of the median MI scores of simultaneously recorded place cell and grid cells per trial (0.34 ± 0.05 for place cells, 0.72 ± 0.05 for grid cells, *t*(15) = −5.66, *p* < 0.001, average difference of medians: 0.38 ± 0.07). **m** Median population vector MI scores between place cell and grid cell firing patterns (median = 0.11, IQR = 0.26 for place cells, median = 0.63, IQR = 0.24 for grid cells, Wilcoxon rank sum test, *z* = −5.17, *p* < 0.001; dashed lines cover *q*_1_ − 1.5 × IQR to *q*_3_ + 1.5 × IQR including all data points)

Figure 4 shows grid cell firing patterns under visual gain decrease (*G* = 2/3). Grid scale and firing patterns show an even stronger weighting towards motor influence than for gain increase trials (median MI score = 0.89, IQR = 0.30 for firing rate maps, Fig. 4i, and median = 0.71, IQR = 0.26 for population vectors, Fig. 4j), with grid scale along the manipulated axis unchanged when plotted in motor coordinates and decreased when plotted in visual coordinates (because a given visual distance reflects greater physical distance, Fig. 4e, f). It is possible that increasing visual gain increases the salience of (now increased) optic flow, and vice versa when decreasing gain, explaining the greater visual influence during gain increase (Fig. 3), discussed below.

### Analysis of individual firing fields, and running speed

Changes in the shapes of individual grid firing fields under gain decrease show the opposite effect to the scale of grid patterns (field size increased along the manipulated axis when plotted in visual coordinates, Fig. 4g, while grid scale decreased, Fig. 4e), giving broader firing fields in probe trials plotted in visual coordinates (Fig. 4c, S1-2). If grid cell firing locations are reset by environmental inputs in disparate locations^22–24^, but otherwise strongly driven by motor inputs, changes to gain will cause offsets between firing locations when running in opposing directions along the manipulated axis. This directional-offset effect was present in grid cell firing during visual gain decrease, explaining the broader firing fields in whole-trial firing rate maps, and more so than in gain increase trials perhaps because of the greater visual influence during gain increase (Supplementary Figure 8). The directional modulation of firing of place cells (but not grid cells) in VR precluded a similar directional-offset analysis of place fields, see Supplementary Figure 8 and ref. ^21^.

The analysis of individual fields also allowed us to assess the dependence of motor influence score on distance to the nearest environmental boundary, but we found no significant effect in place or grid fields (Supplementary Figure 9). However, virtual boundaries are entirely visual and proximity to physical boundaries in real environments may have greater influence on firing^22^. We also recorded cells in mEC with spatially modulated firing that was not grid-like, see Methods. These spatial cells showed motor influence scores similar to grid cells, but with lower scores in gain decrease conditions (a little more like place cells), see Supplementary Figure 10.

During natural foraging, the animal’s running speed is reflected in increases in the frequency of theta rhythmicity in the local field potential^25^, and in increases in the firing rates of spatial cells^26,27^—most clearly seen in speed cells, by definition^28^. When running along the manipulated axis, there were effects of increasing or decreasing the visual gain on the speed dependence of LFP theta frequency and firing rates compared to the un-manipulated axis (which controls for non-specific changes in theta frequency). The effects on LFP theta frequency speed dependence (i.e. the slope of the plots in Supplementary Figure 11) are as expected from the animal’s perception of speed reflecting visual input in addition to physical motion, while effects on firing rates were qualitatively similar but noisier and not significant.

## Discussion

By changing the gain of the movement of the visual projection in VR relative to the physical motion of the mouse, we have shown that the 2-d spatial firing patterns of CA1 place cells are more strongly influenced by visual inputs whereas those of grid cells show a greater influence of physical motion (i.e. proprioception and motor-efference). This difference is seen in the motor influence (MI) scores for place and grid cell firing patterns individually, as population vectors and in simultaneously recorded populations (Fig. 4k–m), see Figures S6 and S7 for the distributions within each animal and Supplementary Figure 9 for similar analyses comparing place and grid firing fields. We also investigated the spatial scale of grid cell firing patterns along the unchanged dimension of the VR, but did not observe any changes (*n* = 107, *t*(106) = −0.15, *p* = 0.88, Figs. 3 and 4), ruling out significant non-spatial effects of the between-trials gain manipulation such as surprise or uncertainty.

The VR system may under-estimate the influence of physical motion, by omitting vestibular cues to linear acceleration which contribute to speed perception and LFP theta frequency^21,29,30^. It also does not include the usual uncontrolled local environmental cues, resulting in more directional place cell firing in VR than in the real world^21,31^. The balance of influence of physical motion and environmental sensory inputs may change according to conditions (e.g. in darkness or on linear tracks, where physical motion may be more influential^17^). In addition, the influence of vision on firing may be enhanced by the foraging task in which mice must pay attention to the (randomly scattered) visual reward pillars.

Nonetheless, our results clearly indicate a significant influence of environmental visual inputs, when available, on place and grid cell firing patterns, and a relatively greater influence on place than grid cell activity, which cannot reflect uncontrolled cues outside of the VR. These differences also show that the firing of place and grid cell populations are not tightly coupled. The lack of overlap in the median MI scores of simultaneously recorded place and grid cells (Fig. 4l), and in the population vectors per animal (Supplementary Figure 7), indicates that neither population’s representation of space is derived solely from the other.

The wide spread of MI scores across the place cells recorded in each animal (Supplementary Figure 6), though not in the overall population vectors (Figs. 1i, j and 2g, h), is consistent with the wide range of effects on individual place cells’ firing of similar manipulations on 1-d virtual tracks^17^. This indicates variation in the balance of inputs received by individual place cells. It is possible that the balance of inputs varies with a cell’s location within CA1, as locations proximal to CA3 receive more input from medial (cf. lateral) entorhinal cortex, i.e. potentially more grid cell mediated self-motion information^32,33^. However, there is only a single CA1 implant in each animal, and the correlation between implant location and average MI scores does not reach significance (Supplementary Figure 12). The depth of the cell within the layer may also be a factor, as this has been shown to correlate with the influence of individual landmarks on place cell firing^34^.

The partial influence of visual inputs on grid cell firing patterns was seen more strongly during increases in visual gain than during decreases (Figs. 3 and 4; median = 0.58, IQR = 0.21 for MI scores in gain increase, median = 0.89, IQR = 0.30 for MI scores in gain decrease, Wilcoxon rank sum test, *z* *=* −5.25*, p* *<* 0.001). Increased (cf. decreased) visual motion signals may have greater salience in a grid system driven by motion rather than environmental features, due to their increased amplitude compared to (unchanged) physical motion signals. This finding is consistent with the effects of gain manipulations on grid cells’ firing patterns on 1-d virtual tracks^18^, in which grid scale shows a greater influence of vision under increased visual gain than decreased visual gain. The 1-d study also suggests that discrete landmarks along the track can reset grid phase if their discrepancy with self-motion is small enough, consistent with attractor models in which intrinsic grid scale reflects physical self-motion^18^. Future work might consider the resetting of grid firing patterns in open arenas, and this occurs specifically at the boundary^22^, or whether distal landmarks also influence grid firing within the arena, possibly mediated by place cells^5^, consistent with effects of hippocampal inactivation^35^, or via other means of overcoming parallax^36^.

Direction-dependent offsets of grid patterns produced broadened firing fields in whole-trial firing rate maps under gain decrease while the underlying grid pattern remained in proportion (Supplementary Figure 8). This may reflect the strong motor influence on grid firing during decreased visual gain, combined with occasional sensory resetting, and could relate to direction-dependent offsets seen in place and grid firing in real environments^37,38^. These results support the regularity of grid cell firing patterns as reflecting an intrinsic metric based on self-motion^2,5–7^, while place cell populations are more strongly driven by sensory environmental inputs^11,12^.

The effect of the gain manipulations on the relationship between running speed and LFP theta frequency indicate a weak influence of vision (Supplementary Figure 11, possibly weakened because trajectories include running along the non-manipulated axis). Slightly stronger visual influences on theta frequency, and on speed cell firing, were seen on the 1-d virtual track^18^, and were greater under gain increase than gain decrease, echoing the visual influence on grid cell firing. However, the much stronger effects on grid scale suggest that they do not simply reflect those on speed coding.

Self-motion signals can be used to guide behaviour (often referred to as ‘path integration’). Studies using 1-d VR show that mice remember the rewarded distance along a track using self-motion cues and that this is impaired by inactivation of stellate cells in mEC^39^. In addition, the remembered distance is influenced by visual inputs in a similar way to grid cell firing—with greater visual influence during gain increase than gain decrease^18^. These results, and the difference between grid and place cell coding of location found here, suggest that grid cell firing may influence spatial memory directly (see also ref. ^40^) rather than acting via place cells.

In summary, although place and grid cell firing patterns are likely to be oriented by similar head-direction information^2,11^, and to communicate with each other to produce a combined estimate of location^5–7^, our results indicate that each cell type receives influential inputs independent of the other, and that these are weighted towards environmental sensory inputs for place cells and towards physical motion-related inputs for grid cells. These results imply corresponding differential roles for place and grid cells in representing evidence for states and for transitions in conceptual spaces^4,6,10^.

## Methods

### Virtual reality

The virtual reality system is presented in ref. ^21^, the virtual reality and surgical methods sections are reproduced here for convenience, with permission.

A circular head-plate made of plastic (Stratasys Endur photopolymer) is chronically attached to the skull, with a central opening allowing the implant of tetrodes for electrophysiological recording (see the section Surgery). The head-plate makes a self-centring joint with a holder mounted in a bearing (Kaydon reali-slim bearing KA020XP0) and is clipped into place by a slider. The bearing is held over the centre of an air-supported Styrofoam ball. Four LCD screens placed vertically around the ball and two projectors onto a horizontal floor provide the projection of a virtual environment. The ball is prevented from yaw rotation to give the mouse traction to turn and to prevent any rotation of the ball about its vertical axis, following ref. ^20^ (see Fig. 1a, b).

The virtual environment runs on a Dell Precision T7500 workstation PC running Windows 7 64-bit on a Xeon X5647 2.93 GHz CPU, displayed using a combination of four Acer B236HL LCD monitors mounted vertically in a square array plus two LCD projectors (native resolution 480 × 320, 150 lumens) mounted above to project floor texture. The head-holder is at the centre of the square and 60 mm from the bottom edge of the screens, and 9500 mm below the projectors. The LCD panels are 514 mm × 293 mm, plus bezels of 15 mm all around. These six video feeds are fed by an Asus AMD Radeon 6900 graphics card and combined into a single virtual display of size 5760 × 2160 px using AMD Radeon Eyefinity software. The VR is programmed using Unity3d v5.0.2f1 which allows virtual cameras to draw on specific regions of the virtual display, with projection matrices adjusted (see Kooima, 2008, http://csc.lsu.edu/~kooima/articles/genperspective/index.html) to the physical dimensions and distances of the screens and to offset the vanishing point from the centre. For example, a virtual camera facing the X-positive direction renders its output to a portion of the virtual display which is known to correspond to the screen area of the physical monitor facing the X-negative direction.

Translation in the virtual space is controlled by two optical mice (Logitech G700s gaming mouse) mounted with orthogonal orientations at the front and side of a 200 mm diameter hollow polystyrene sphere, which floats under positive air pressure in a hemispherical well. The optical mice drive X and Y inputs, respectively, by dint of their offset orientations, and gain can be controlled within the Unity software. Gain is adjusted such that real-world rotations of the sphere are calibrated so that a desired environmental size (e.g. 600 mm across) corresponds to the appropriate movement of the surface of the sphere under the mouse (i.e. moving 600 mm, or just under one rotation, on the sphere takes the mouse across the environment). Mouse pointer acceleration is disabled at operating system level to ensure movement of the sphere is detected in a linear fashion independent of running speed.

The mouse is able to freely rotate in the horizontal plane, which has no effect on the VR display (but brings different screens into view). Rotation is detected and recorded for later analysis using an Axona dacqUSB tracker which records the position of two LEDs mounted at ~25 mm offset to left and right of the head stage amplifier (see the section Surgery). Rotation is sampled at 50 Hz by detection of the LED locations using an overhead video camera, while virtual location is sampled and logged at 50 Hz.

Behaviour is motivated by the delivery of milk rewards (SMA, Wysoy) controlled by a Labjack U3HD USB Data Acquisition device. A digital-to-analogue channel applies 5 V DC to a control circuit driving a 12 V Cole-Parmer 1/16″ solenoid pinch valve, which is opened for 100 ms for each reward, allowing for the formation of a single drop of milk (5 μL) under gravity feed at the end of a 1/32″ bore tube held within licking distance of the animal’s mouth.

Control of the Labjack and of reward locations in the VR is via UDP network packets between the VR PC and a second experimenter PC, to which the Labjack is connected by USB. Software written in Python 2.7 using the Labjack, tk (graphics) and twistd (networking) libraries provide a plan-view graphical interface in which the location of the animal and reward cues in the VE can be easily monitored and reward locations manipulated with mouse clicks (see Fig. 1 and Supplementary video).

Although the virtual environment had a hexagonally tiled floor (Supplementary Figure 13), grid cell firing patterns are hexagonal on a much larger scale and are not responding to floor tile vertices (Supplementary Figure 14).

### Surgery

Nine C57Bl/6 wild type mice were used (Table 1). Throughout surgery, mice were anesthetized with 2–3% isoflurane in O_2_. Analgesia was provided pre-operatively with 0.1 mg/20 g Carprofen, and post-operatively with 0.1 mg/20 g Metacam. Custom-made head plates were affixed to the skulls using dental cement (Kemdent Simplex Rapid). Mice were implanted with custom-made microdrives (Axona, UK), loaded with 17 μm platinum-iridium tetrodes, and providing buffer amplification. One mouse was implanted with eight tetrodes in CA1 (ML: 1.8 mm, AP: 2.1 mm posterior to bregma), two mice with eight tetrodes in the dorsomedial entorhinal cortex (dmEC, ML = 3.1 mm. AP = 0.2 mm anterior to the transverse sinus, angled 4° posteriorly), and five mice received a dual implant with one microdrive in right CA1 and one in left dmEC (each mircrodrive carried four tetrodes). After surgery, mice were placed in a heated chamber until fully recovered from the anaesthetic (normally about 1 h), and then returned to their home cages. Mice were given at least 1 week of post-operative recovery before cell screening and behavioural training started.

**Table 1:** Manipulation protocol

Mouse id	Implant type^a^	Number of grid firing patterns recorded^b^	Number of place firing patterns recorded^b^	Gain increase (*G* = 2)^c^	Gain decrease (*G* = 2/3)^c^	Number of grid patterns per session^d^	Number of place patterns per session^d^
				Baseline: 60 × 60 cm Probe: 30 × 60 cm	Baseline: 60 × 60 cm Probe: 90 × 60 cm		
864	E	2(2)	0	1	0	2.0	0
984	C	0	21(12)	1	1	0	6.0 ± 0
986	E	17(17)	0	1	0	17.0	0
987	CE	29(29)	83(48)	2(1)	1	9.7 ± 2.8	24.0 ± 3.0
1014	CE	7(7)	0	1	1	3.5 ± 0.5	0
1015	CE	3(3)	53(27)	1	1	1.5 ± 0.5	13.5 ± 3.5
1060	CE	6(6)	38(30)	2	1	2.0 ± 0	10.0 ± 0.6
1061	CE	43(42)	178(105)	3	2	8.4 ± 1.6	21.0 ± 2.5
1176	CE	15(12)	124(53)	2	2	3.0 ± 0.7	13.3 ± 0.6

^a^E: implant in left mEC; C: implant in left CA1; CE: implant in right CA1 and left mEC

^b^Numbers in brackets indicate compact non-remapping firing patterns

^c^Number of whole-day recording sessions for each type of manipulation, environment size given in motor coordinates. Brackets indicate one session without compact non-remapping place firing patterns

^d^Mean ± SEM of number of compact non-remapping place and grid firing patterns analysed per session per animal

### Electrophysiology

Following recovery, mice were food restricted to 85% of their free-feeding body weight. They were then exposed to a recording arena every day (20 min per day) and screening for neural activity took place. The recording arena was a 60 × 60 cm square box placed on a black Trespa ‘Toplab’ surface (Trespa International B.V., Weert, Netherlands), and surrounded by a circular set of black curtains. A white cue-card (A0, 84 × 119 cm), illuminated by a 40 W lamp, was the only directionally polarising cue within the black curtains. Milk (SMA Wysoy) was delivered as drops on the floor from a syringe as rewards to encourage foraging behaviour. Tetrodes were lowered by 62.5 μm each day, until grid or place cell activity was identified, in dmEC or CA1, respectively. Neural activity was recorded using DACQ (Axona Ltd., UK) while animals were foraging in the square environment. For further details see ref. ^19^.

### Behavioural training

In general, behavioural training in VR started as tetrodes were approaching target areas. It involved three phases. Firstly, mice experienced an infinitely long 10 cm-wide virtual linear track, with 5 μL milk drops delivered as rewards. The aim of this training phase was to habituate the mice to being head restrained and train them to run smoothly on the air-cushioned ball. It took three days, on average, for mice to achieve this criterion and move to the next training phase. During the second training phase mice experienced a similar virtual linear track. During this phase, reward beacons were evenly spaced along the long axis of the track, as before, but placed pseudo-randomly in one of three pre-defined positions on the lateral axis (middle, left or right). The aim of this training phase was to strengthen the association between rewards and virtual beacons, and to train animals to navigate towards rewarded locations via appropriate rotations on top of the ball. This training phase also took three days, on average. During the third training phase mice were introduced into a virtual square arena placed in the middle of a larger virtual room. The virtual arena in the third training phase had size 60 × 60 cm or 90 cm × 90 cm for different mice. Mice were trained on a ‘random foraging’ task, during which visible beacons were placed in the square box at random locations (at any given time only one beacon was visible), and a ‘fading beacon’ task, during which animals had to learn to navigate to a fixed location for invisible rewards (see ref. ^21^ for details).

### Manipulating the gain of visual motion vs physical motion

After the animals had been trained in the ‘fading beacon’ task, they ran in a 60 × 60 cm VR square (Supplementary Figure 13) as their baseline environment. A probe session consisted of a 40-min random-foraging baseline trial followed by a 40-min random-foraging probe trial and a final 20-min real-world (R) trial. In the probe trial, the ball-movement to visual-movement gain setting on one of the axes was either double for the gain increase manipulation (gain 'G'  = 2; i.e. animals had to run half of the distance on the ball to move the same distance in the visual VR on the manipulated axis compared to the baseline trial) or 2/3 for the gain decrease manipulation (gain 'G'  = 2; i.e. animals had to run 1.5× the distance on the ball to cover the same distance in the visual VR on the manipulated axis compared to the baseline trial).

The location in virtual environments can be plotted in motor coordinates or visual coordinates. In baseline trials (where Gain = 1) these locations and the size of the virtual environment is the same in both coordinates. In probe trials, where the ball-to-vision gain changed (Gain = 2 or 2/3), environment size is different to the 60 × 60 cm baseline when measured in motor coordinates (i.e. 30 × 60 cm or 90 × 60 cm) but is the same in visual coordinates. We did not use Gain = 1/2 probe trials, as large environments—120 cm or more across in motor coordinates—become impractical in terms of getting good coverage and also produce less stable grid cell firing patterns near to the centre, presumably reflecting the absence of local cues and large distance and low parallax of visual cues to location.

### Data analysis

Spike sorting was performed offline using an automated clustering algorithm (KlustaKwik) followed by a manual review and editing step using an interactive graphical tool (waveform, Daniel Manson, http://d1manson.github.io/waveform/). After spike sorting, firing rate maps were constructed by binning animals’ positions into 1.5 × 1.5 cm bins, assigning spikes to each bin, smoothing both position maps and spike maps separately using a 5 × 5 boxcar filter, and finally dividing the smoothed spike maps by the smoothed position maps.

In VR probe trials, relative virtual/visual positions were different from relative physical positions on the ball due to the changing gain between visual movement and physical movement. Rate maps in a visual coordinate were constructed by binning animal’s virtual (visual) locations. Then rate maps in a motor coordinate were reconstructed by linear interpolation of the existing rate maps according to the applying gain ratio.

Cells were classified as place cells if their spatial information in the baseline trial exceeded the 99th percentile of a 1000 shuffled distribution of spatial information scores calculated from rate maps where spike times were randomly offset relative to position by at least 4 s. Cells were classified as grid cells if their gridness scores in the baseline trial exceeded the 99th percentile of a shuffled distribution of 1000 gridness scores, following refs. ^27,41^. For each shuffle, a fixed time offset was added to all spike times for each cell, with spike times after the end of the trial ‘wrapped’ to the beginning of the trial. Position data was unchanged. In this way, the temporal dynamics of the spike train were preserved, but the relation of the spikes to position was uncoupled. Each cell was compared to its own individual shuffled data. To check that grid-like responses were not generated by visual input from the hexagonal floor tiles we plotted the scales and phases of grid cell firing patterns relative to the floor pattern—finding no correspondence (see Supplementary Figure 14).

Speed-modulated cells were classified from the population of the recorded cells in baseline trials, following ref. ^27^. Briefly, the degree of speed modulation for each cell was characterised by first defining the instantaneous firing rate of the cell as the number of spikes occurring in each position bin divided by the sampling duration (0.02 s). Then a linear correlation was computed between the running speeds and firing rates across all position samples in a trial, and the resulting *r*-value was taken to characterise the degree of speed modulation for the cell. To be defined as speed-modulated, the *r*-value for a cell had to exceed the 99th percentile of a distribution of 1000 *r*-values obtained from spike shuffled data.

Cells in mEC were defined as spatial non-grid cells, using similar criteria to place cells, if their spatial information in the baseline trial exceeded the 99th percentile of the shuffled distribution of spatial information and their gridness scores in the baseline trial did not exceed the 99^th^ percentile of the shuffled gridness score distribution.

To identify ‘remapping’ as opposed to stretched or offset firing rate maps, we calculated the spatial correlation between a baseline firing rate map and the best-matching (stretched and offset) probe rate map (described above). We defined a remapped cell as having a spatial correlation between the two maps below 0.3. Only non-remapped place cells and grid cells were included in further analysis, leaving 425 out of 497 place cells and 118 out of 122 grid cells that were quantified as non-remapping cells. The proportions of place cells remapping between baseline and probe conditions did not differ between gain increase and gain decrease sessions (Supplementary Figure 5).

For place cells, we then identified firing fields at 30% peak firing rates, and considered those cells whose fields covered more than 50% of either probe or baseline environments as too diffuse for analyses, leaving 275 out of 497 compact non-remapping place cells for further analysis.

To compare grid scale changes along different axes, we fitted an ellipse circumscribing the six peaks nearest to the centre on the autocorrelogram of a cell. The grid was not included in the analysis if the number of nearest peaks was less than six. We then measured the diameters of the ellipse along manipulated and un-manipulated axes. The ratios of the diameters in probe trials to those in baseline trials were calculated based on the autocorrelograms generated in visual coordinates and motor coordinates.

To determine the ‘motor influence score’ for firing patterns, a set of 20 transformed probe trial firing rate maps were generated via linearly interpolating firing rate maps plotted in a visual coordinate by stretch factors ranging from 1 to the applied gain ratio in 20 steps. Correlations between the transformed probe maps and baseline rate maps were then calculated with offsets of 1.5 cm intervals along the manipulated axis between the smaller to the larger map, keeping the largest^12^. A stretch factor (*F*) was defined as the factor that produced the largest spatial correlation. The visual gain *G* is the ratio distance moved by the viewpoint in VR divided by the distance moved by the mouse on the ball surface. The ‘motor influence’ score was defined as MI = (*F*−1)/(*G*−1) for the probe trials, so that MI = 1 if the stretch factor is equal to the visual gain (i.e. *F* = *G*) and MI = 0 if the stretch factor is unity despite non-unity visual gain (*F* = 1, *G* ≠ 1).

### Motor influence score for population vectors

To determine the ‘motor influence score’ for population vectors, for each session containing more than one cell, we computed one population vector. First, we combined all simultaneously recorded place cells/grid cells in a vector. For each cell, we calculated the spatial correlation values between the baseline rate map and the transformed probe map with stretch factors ranging from 1 to the applied gain ratio in 20 steps. Then we calculated the average spatial correlation values over all cells for each stretch factor, with offsets of 1.5 cm intervals along the manipulated axis between the smaller to the larger map. The offset and stretch factor for the population vector (Op, Fp) was defined as the pair with the highest average correlation over cells. The motor influence score for the population vector was defined as MIp = (Fp−1)/(*G*−1).

To compare firing field size changes along different axes, we defined firing fields based on the rate maps of the cells. For each rate map, we first excluded the bins with firing rates lower than 30% of peak rates. Then firing fields were found by fitting ellipses around sets of contiguous bins containing firing. Fields which were smaller than 5% or bigger than 50% of the total area were excluded in further analysis.

To find matching fields in the probe trial, we calculated the centroid position C(*x*,*y*) for each field in the baseline trial. We then calculated the matching centroid in a probe trial by shifting the *x* position of the baseline centroid by the best fitting stretch factor *F*. A matching field was defined as a field which included the matching centroid.

We excluded grid fields near the edge of the environment as they appear to be occluded by the edges—causing significant changes in field shape between baseline and probe trials (e.g. circular to semi-circular). Perhaps because they are largely motor driven, environmental edges simply prevents further sampling of grid field. By contrast, place fields near to the edge seem to retain their location and shape (albeit potentially stretched), perhaps because they are largely driven by vision (and, indeed, the distance to the boundary^5^). Place fields were processed similarly to grid fields for ease of comparison (i.e. setting the rate map outside of the field to zero and excluding place fields near to the edge).

### Motor influence score for individual firing fields

To determine the ‘motor influence score’ for individual firing fields, for each field that we identified with the method described above, we constructed a new rate map including the field and zero spikes for the remaining position bins. Then we computed the motor influence score from the rate map constructed for that field.

### Ethical compliance

All procedures were performed in accordance with UK Home Office project license 70/8636 to F.C.

## Supplementary information

Supplementary Information

Supplementary Movie 1

Description of Additional Supplementary Files

## Data Availability

Data are available on Open Science Framework, https://osf.io/uk68w/, with descriptions of the variables, and MatLab scripts available on request.
