# Supplementary material for: Differential influences of environment and self-motion on place and grid cell firing
Source: Nat Commun. 2019 Feb 7;10:630. doi: 10.1038/s41467-019-08550-1 (PMC6367320; doi:10.1038/s41467-019-08550-1)
Supplement: Supplementary file 1 — Supplementary Information [file 41467_2019_8550_MOESM1_ESM.pdf]

Differential influences of environment and self-motion on place and grid cell firing. *Chen et al.*

Supplementary Information

Supplementary Figures

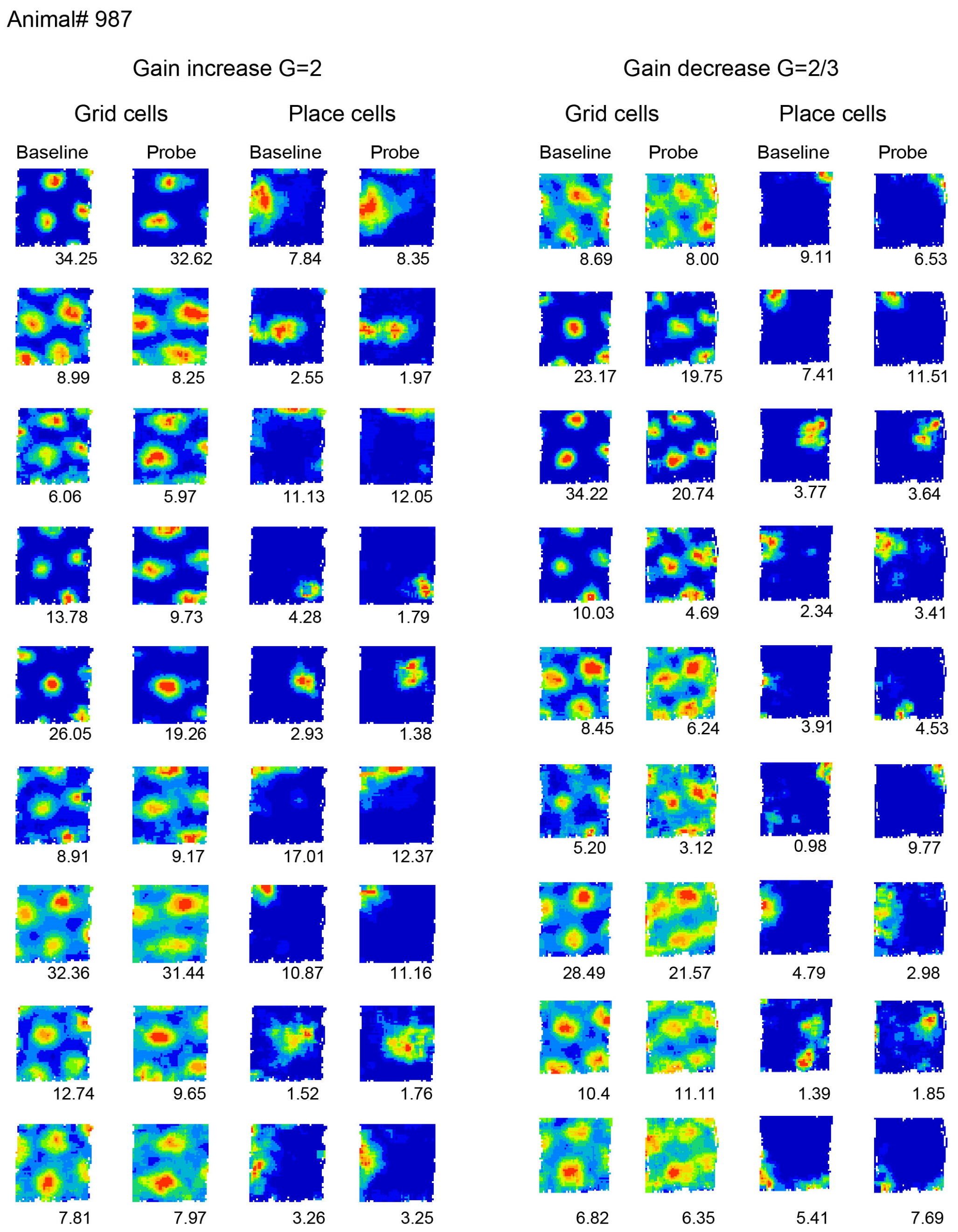

Supplementary Figure 1. Examples of simultaneously recorded place and grid cells. Showing firing rate maps from baseline and probe trials on a gain increase day and on a gain decrease day, with peak rate in Hz below, from mouse 987.

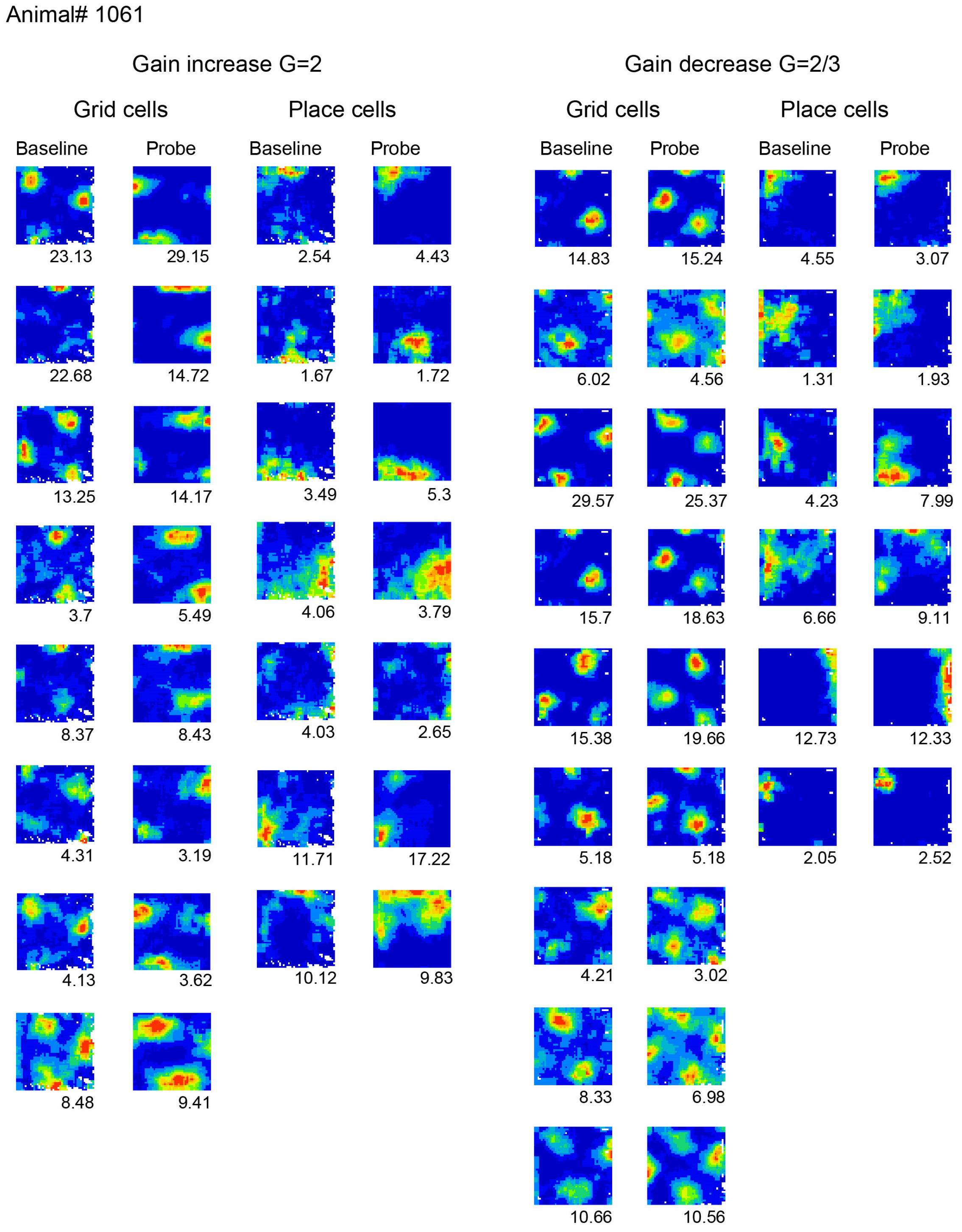

Supplementary Figure 2. Examples of simultaneously recorded place and grid cells. As Supplementary Figure 1, from mouse 1061.

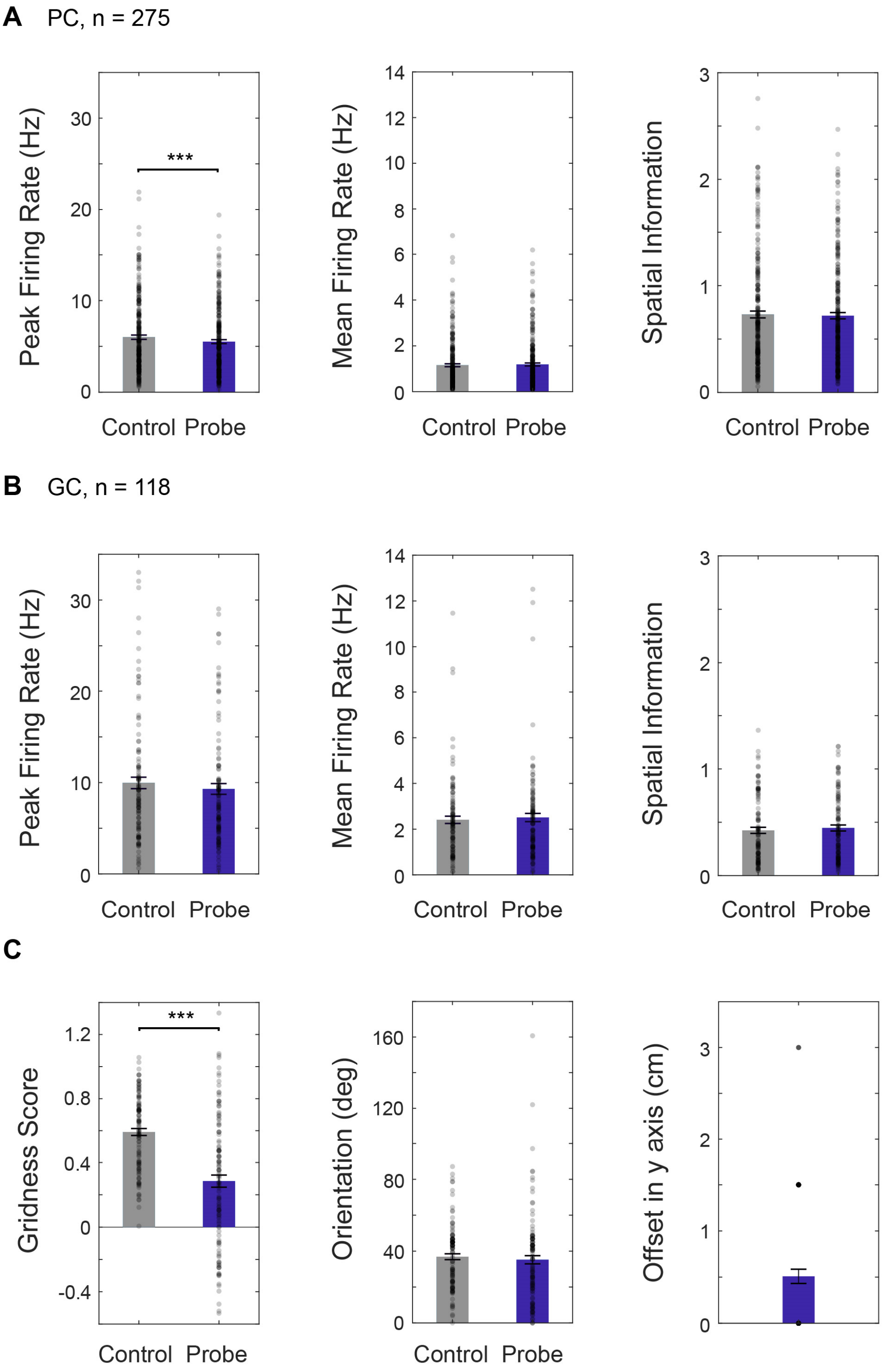

Supplementary Figure 3. Place and grid cell firing properties across baseline and gain trials. (A) Comparison of place cells peak firing rate, mean firing rate and spatial information in VR baseline and probe trials. Peak firing rate is higher in VR baseline than in probe trials ($t(274) = 3.68$, $p < .001$). (B) Comparison of grid cells peak firing rate, mean firing rate and spatial information in VR baseline and probe trials. Peak firing rate is higher in VR baseline than probe trials ($t(117) = 2.42$, $p = 0.02$). (C) Comparison of grid cells gridness score, grid orientation and grid pattern offset in y axis between VR baseline and probe trials. Gridness score is higher in VR baseline than in probe trials ($t(117) = 8.09$, $p < 0.001$). Probe trial analyses are performed on the best fitting stretched rate maps.

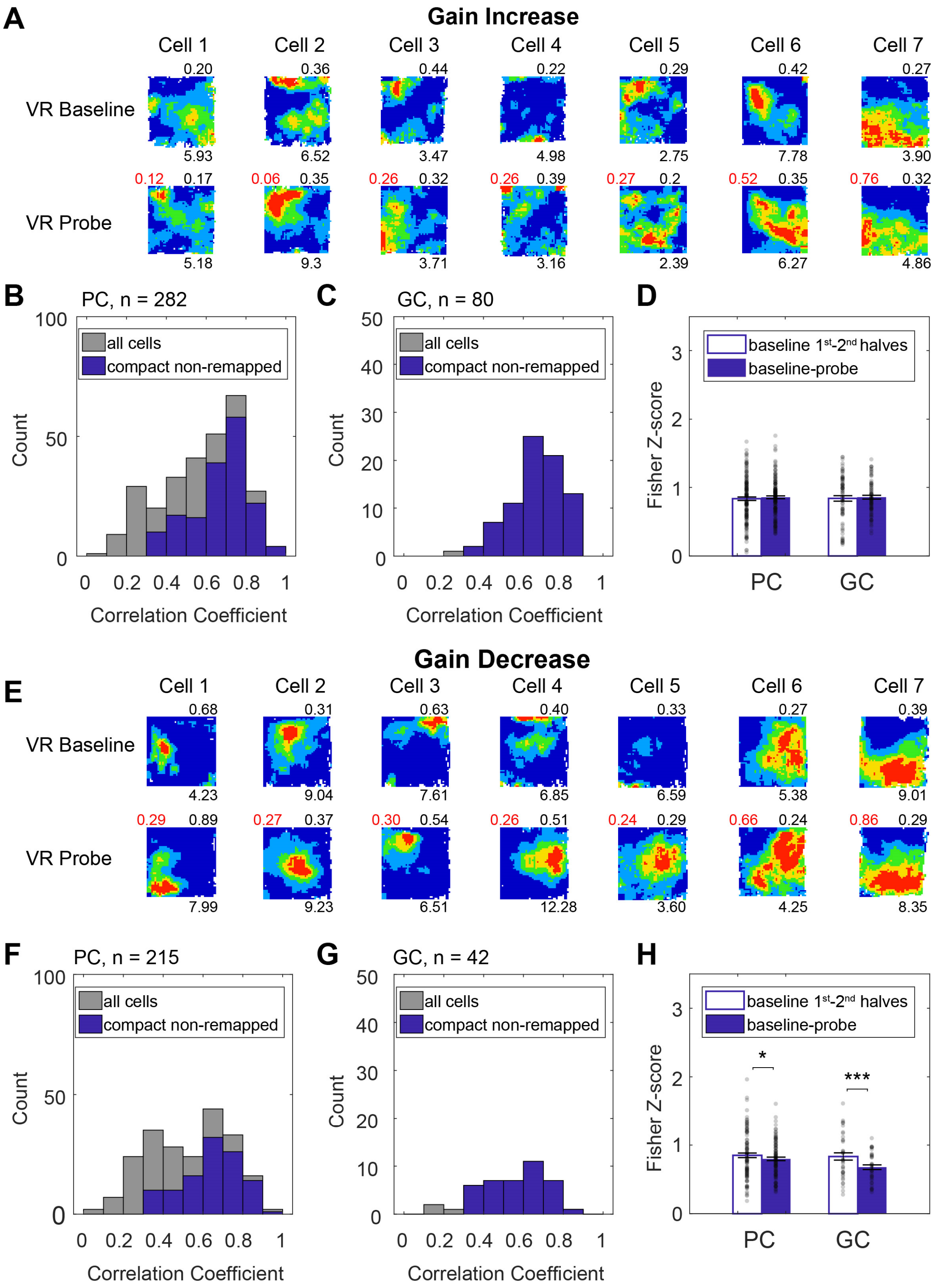

Supplementary Figure 4. Comparison of all place and grid cells to those with compact non-remapping firing patterns. To enter the main analyses, spatial correlations between probe and baseline firing rate maps (taking account of the effect of the gain manipulation) had to exceed 0.3, and place firing patterns had to cover <50% of baseline and probe environments, here we illustrate the properties of the excluded cells relative to the compact non-remapping cells. (A) Five place cells

which remapped in the gain increase probe trial (bottom row, in visual coordinates) compared to the baseline trial (top row) and two place cells which had diffuse fields. Firing rate maps shown with spatial information (bits/spike) in black, correlation coefficients between baseline and best-fitting probe in red (above), and max rates (Hz, below). (B-C) Distribution of correlation coefficients between baseline and best-fitting (stretched and offset) probe rate maps in gain increase trials for place (B) and grid cells (C). (D) Fisher's Z scores of the correlation coefficients between baseline and best-fitting gain increase rate maps were similar to those between 1st and 2nd halves of baseline trials, for both place ($n=166$, $t(165)=-1.05$, $p=0.30$) and grid ($n=79$, $t(78)=-0.63$, $p=0.53$) firing patterns. (E) Five place cells which remapped in the gain decrease probe trial (bottom row, in visual coordinates) compared to the baseline trial (top row) and two place cells which had diffuse fields. (F-G) Distribution of correlation coefficients between baseline and best-fitting probe rate maps in gain decrease trials for place (F) and grid cells (G). (H) Fisher's Z scores of the correlation coefficients between baseline and best-fitting gain decrease rate maps were lower than those between 1st and 2nd halves of the baseline trials, for both place ($n=109$, $t(108)=2.06$, $p<0.05$) and grid ($n=39$, $t(38)=4.37$, $p<0.001$) firing patterns. NB Fisher's Z-transformation of correlation coefficient r, to increase Normality, was: $Z = \frac{1}{2}\ln\left(\frac{1+r}{1-r}\right)$.

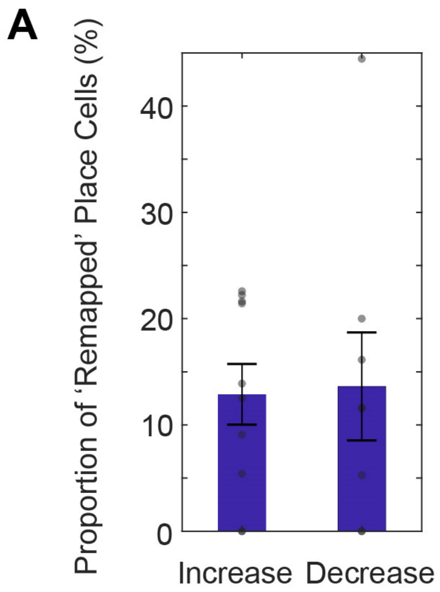

Supplementary Figure 5. Proportion of 'remapped' place cells. There is no difference in the proportion of place cells remapping between baseline and probe trials when comparing gain increase and gain decrease sessions (t(16) = -0.14, p = 0.89).

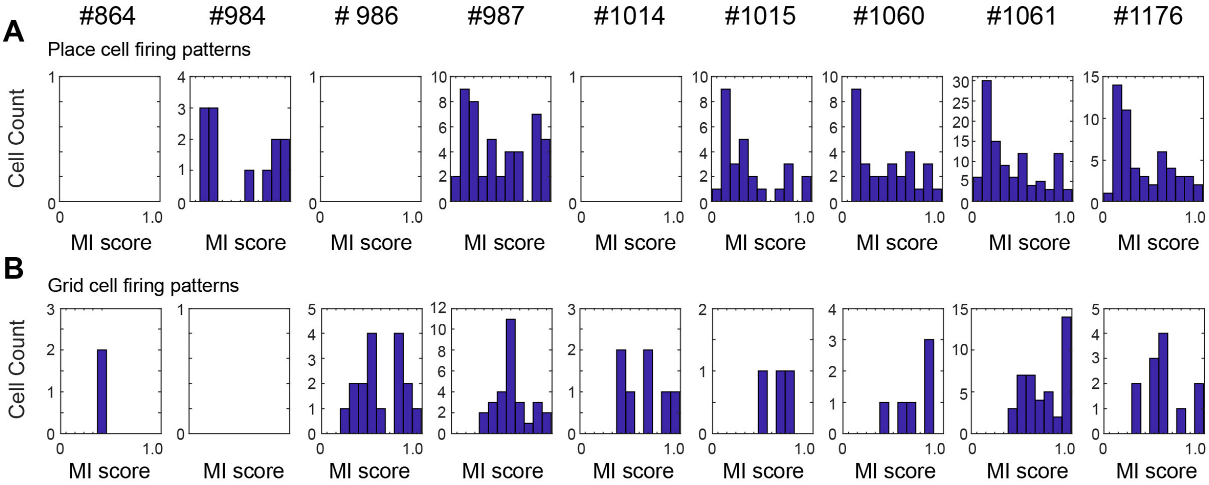

Supplementary Figure 6. Distribution of motor influence score (MI) for place (upper panel) and grid cell firing patterns (lower panel) per animal. Note the overall weighting towards visual influence for place cell firing patterns (Figures 1I, 2G) and to intermediate or motor influence for grid cell firing patterns (Figures 3I, 4I).

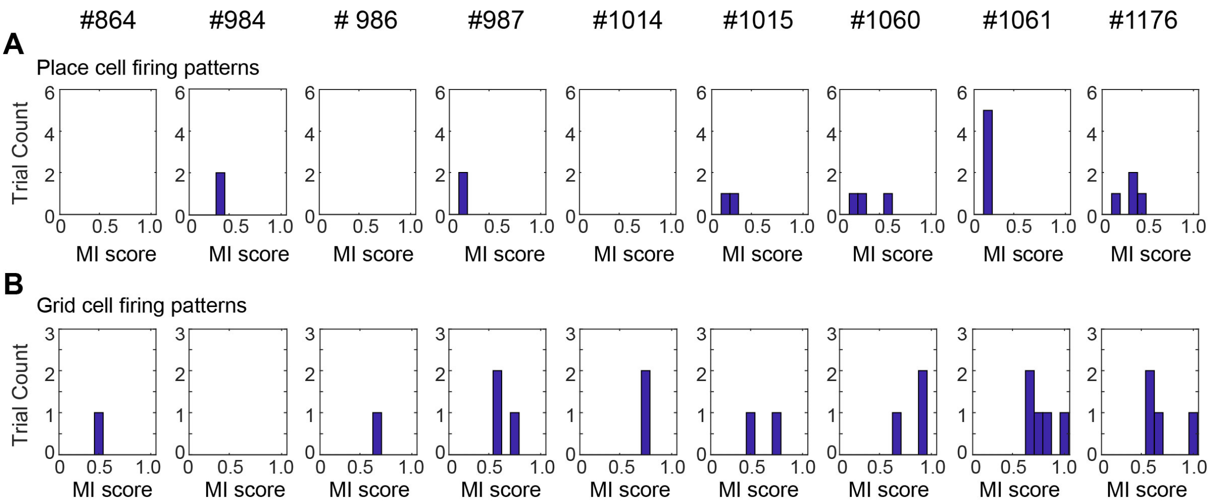

Supplementary Figure 7. Distribution of motor influence scores for population vectors, for place (upper panel) and grid cell firing patterns (lower panel) per animal. Note the overall weighting towards visual influence for place cell population vectors (Figures 1J, 2H) and to intermediate or motor influence for grid cell population vectors (Figures 3J-4J).

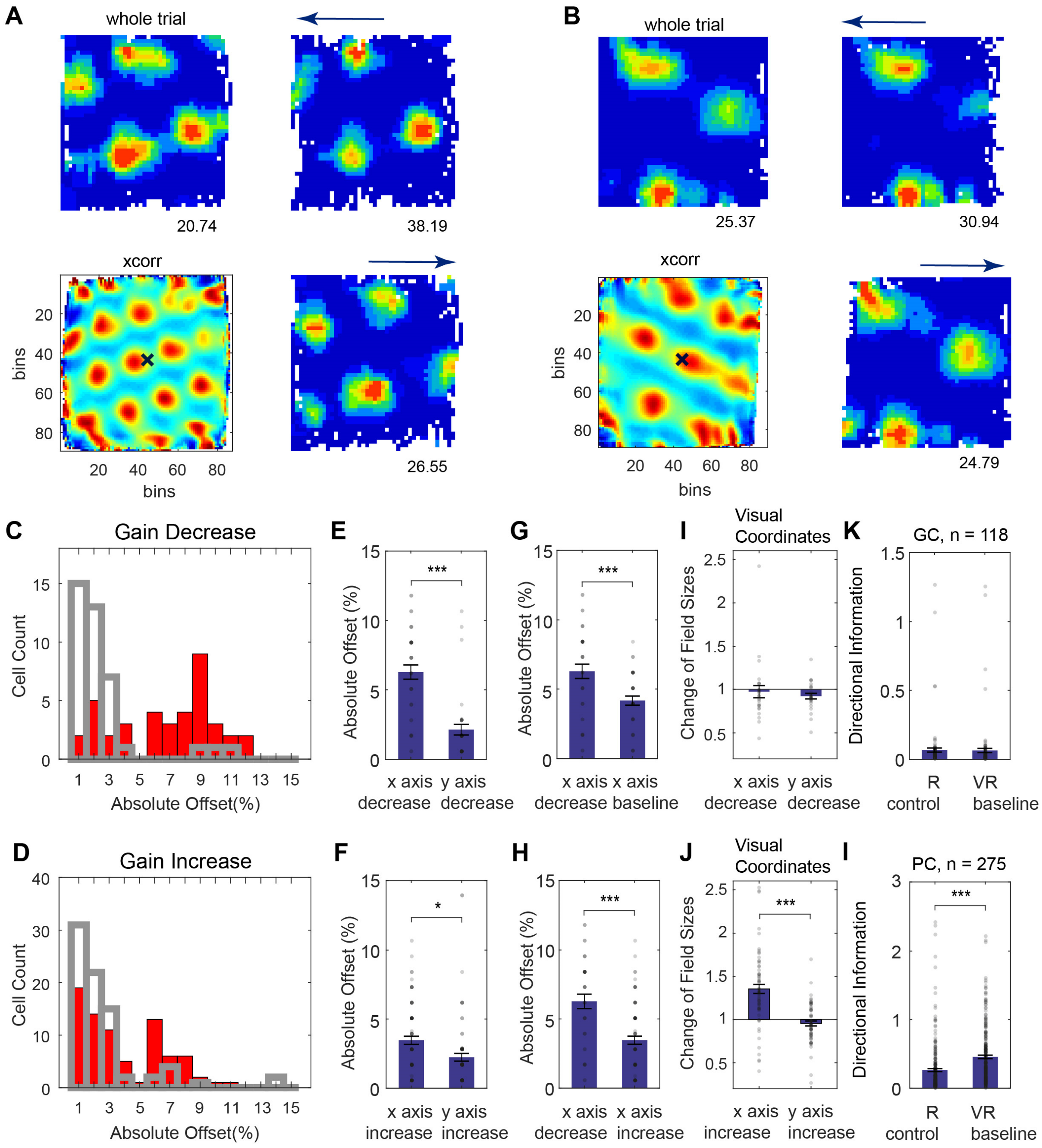

Supplementary Figure 8. Direction dependent offsets of grid patterns. (A) Example grid cell showing grid fields shifting forwards in running direction along the manipulated axis of a gain decrease trial. (B) Example grid cell showing grid fields moving backwards against the running direction along the manipulated axis of a gain decrease trial. Rate maps are shown in visual coordinates for the whole trial (top left); filtered by running direction (arrow above plot, right column, westwards above and eastwards below). Max firing rates shown below the rate maps. The spatial cross-correlogram of the two direction-filtered rate maps (bottom left) shows the offset from the centre (x). (C-D) Distribution of absolute offsets in gain decrease (C) and gain increase (D) trials (red) and their baselines (grey). (E-F) Directional-offsets in probe trials along the manipulated (x) axis were greater than along the non-manipulated (y) axis in gain decrease (E, paired t-test, $t(38)=6.34$, $p<0.001$) and gain increase trials (F, paired t-test, $t(78)=3.08$, $p<0.05$). (G) The offset along the x axis in gain decrease trials was significantly greater than in baseline trials (paired t-test, $t(38)=4.59$, $p<0.001$), or in gain increase trials (H; two sample t-test, $t(116)=5.11$, $p<0.001$). (I) Change of grid field sizes, found separately for

eastward and westward runs and then averaged, was similar on the manipulated (x) and un-manipulated (y) axes in gain decrease trials (t(64)=0.71, p=0.48), unlike Figure 4G. (J) Change of grid field sizes, found separately for eastward and westward runs and then averaged, was significantly greater along the x than y axes in gain increase trials (t(64)=7.72, p<0.001), as in Figure 3G. (K) The directionality of grid cell firing was similar in both R and VR (paired t-test, t(117)=1.12, p=0.27). (L) Place cell firing was significantly more directional in VR than R (paired t-test, t(274)=-10.20, p<0.001), precluding directional offset analyses.

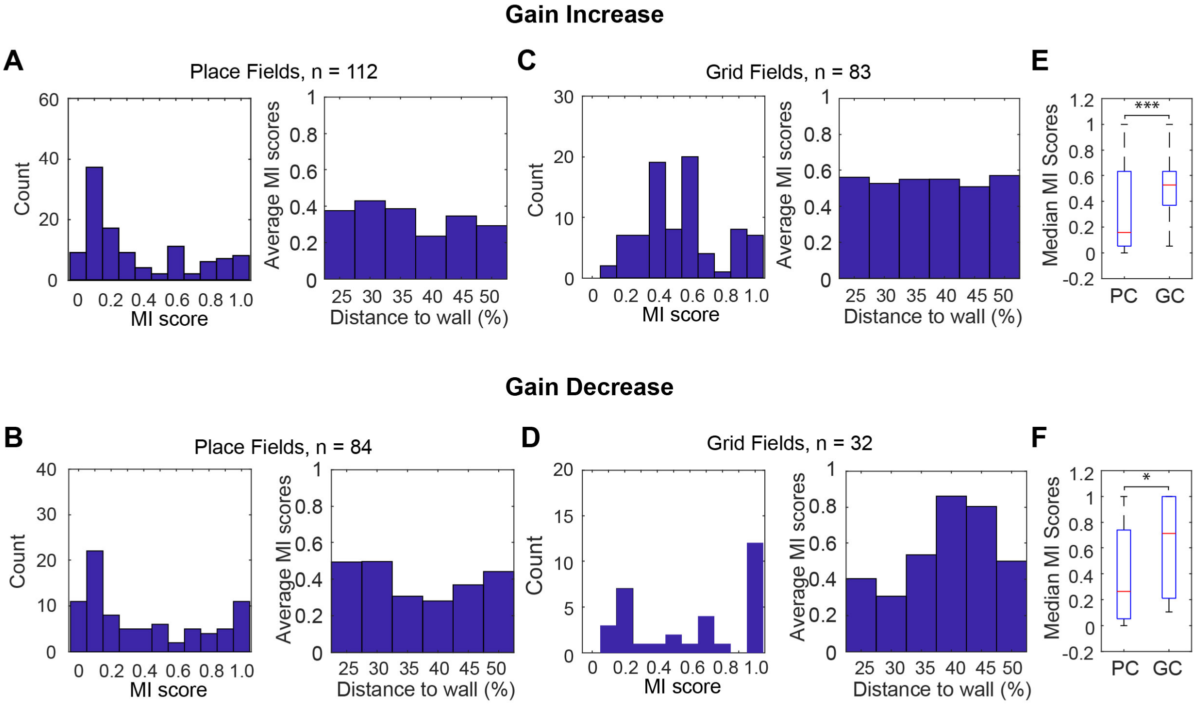

Supplementary Figure 9. Analysis of individual firing fields and distance to nearest boundary. (A-B) Motor influence (MI) distributions for place fields distal to boundaries under gain increase and decrease separately (note similarity to the MI for firing rate maps in Figs 1-2, but here the rate map outside the field is set to zero and only distal fields used, to match the grid field analysis). Distance to nearest boundary had no clear effect on the motor influence (r=-0.62, p=0.18 in gain increase, r=-0.38, p=0.45 in gain decrease). (C-D) Motor influence (MI) distributions for grid fields distal to boundaries under gain increase and decrease separately (note similarity to MI Figures 3-4). Distance to nearest boundary had no significant effect on motor influence (r=-0.01, p=0.97 in the gain increase, r=0.55, p=0.25 in the gain decrease). (E-F) Median motor influence scores of these place fields were significantly different from those of grid fields in both gain increase and gain decrease trials (Wilcoxon rank sum test, z=-5.01, p<0.001 in gain increase, and z=-3.23, p<0.05 in gain decrease).

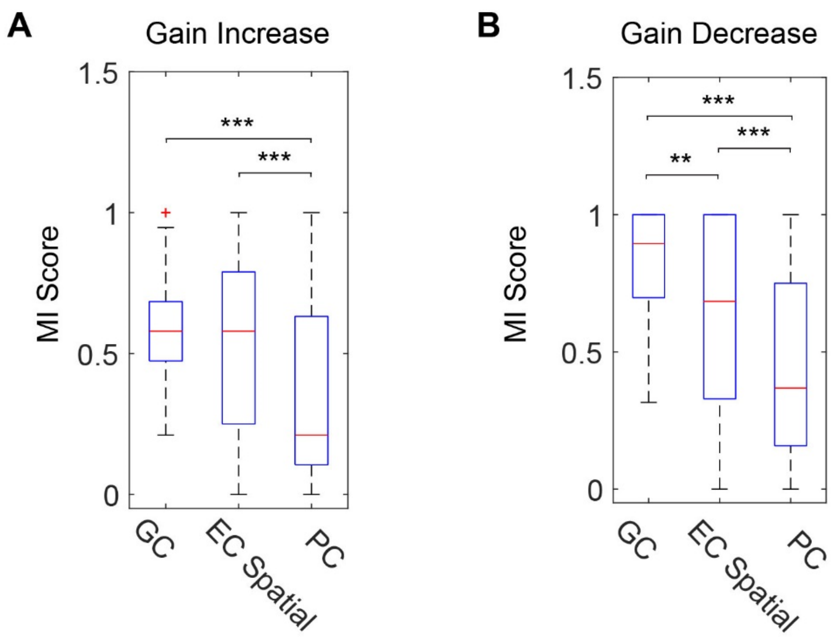

Supplementary Figure 10. Motor influence (MI) scores for spatial cells in mEC. (A) Median MI scores of grid cells, entorhinal spatial cells and place cells in gain increase sessions. Median (red line)=0.58, interquartile range (IQR, blue box)=0.21 for grid cells, Median=0.58, IQR=0.54 for spatial cells, Median=0.21, IQR =0.53 for place cells, Wilcoxon rank sum test between GC and EC spatial cells, z=1.59, p=0.11; between PC and EC spatial cells, z=-4.33, p<0.001; Wilcoxon rank sum test between PC and GC, z=-5.72, p<0.001). (B) Median MI scores of grid cells, entorhinal spatial cells and place cells in gain decreased sessions (Median=0.89, IQR=0.30 for grid cells, Median=0.68, IQR=0.67 for EC spatial cells, Median=0.37, IQR=0.59 for place cells, Wilcoxon rank sum test between GC and EC spatial cells, z=2.74, p<0.01, between GC and EC spatial cells, z=-3.99, p<0.001; Wilcoxon rank sum test between GC and PC, z=-5.79, p<0.001).

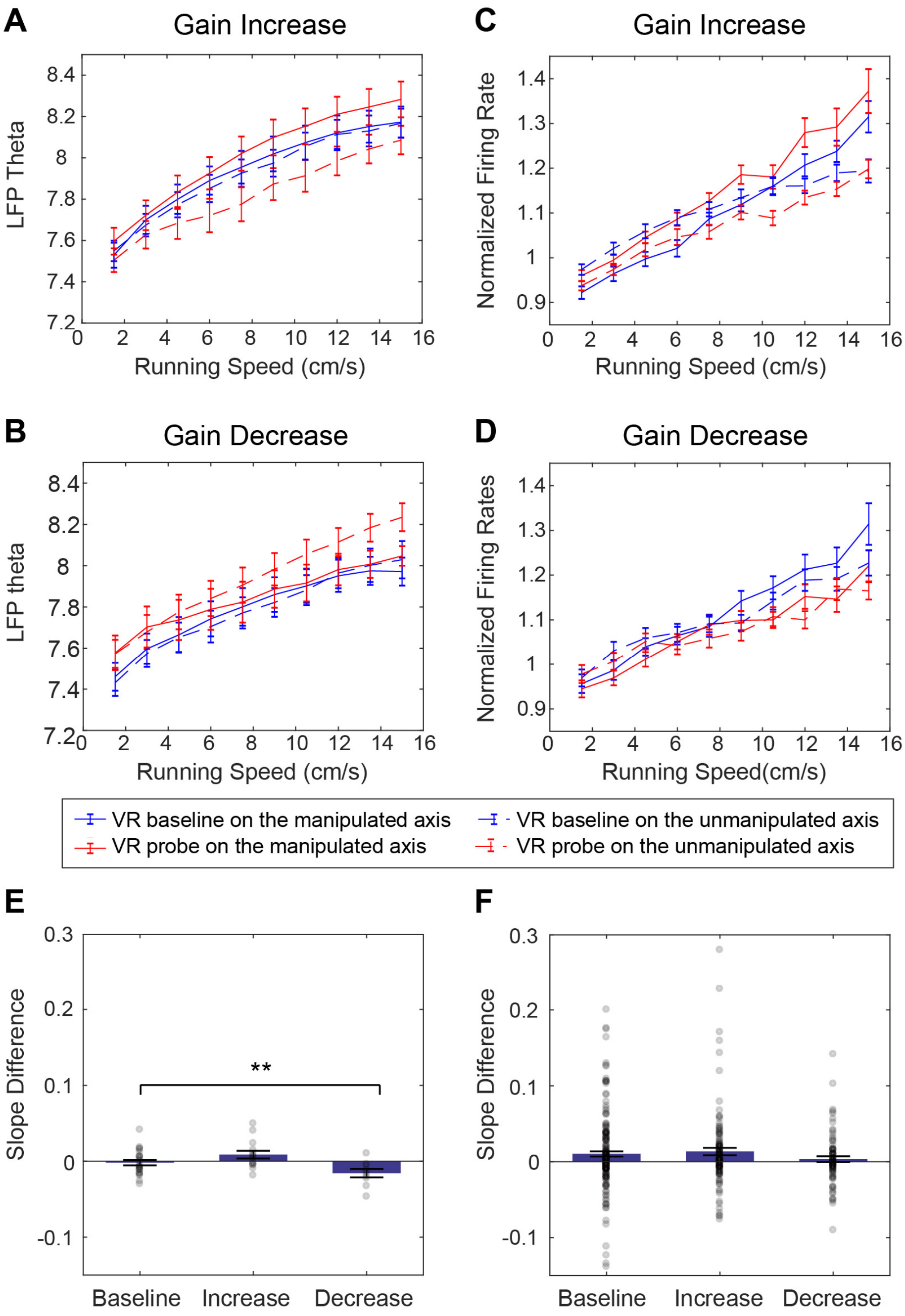

Supplementary Figure 11. LFP theta frequency (A-B) and speed cell firing rates (C-D). Shown as functions of speed of movement along the manipulated (dotted lines) and un-manipulated (solid lines) axes in baseline (blue) and probe (red) trials. Lines show mean (±s.e.m) theta frequency or firing rate (normalised by each cell's overall firing rate) in each running speed bin (1.5cm/s to 15cm/s). (E) The slopes of the LFP theta frequency versus running speed relationship differed between manipulated and un-manipulated axes. This difference showed a significant main effect of gain across increase, decrease and baseline trials (n=45, F(2,43)=5.47, p<0.01). The difference between baseline and gain decrease trials reached significance (n=9, paired t-test, t(8)=2.35, p<0.05), that between baseline and gain increase trials did not (n=14, paired t-test, t(13)=-1.94, p=0.07). (F) The slopes of speed cell firing rate-running speed relationships show some differences between the manipulated and un-manipulated axes but did not show a significant main effect of gain across trials (n=383, F(2,381)=1.10, p=0.33). Slope was calculated as the gradient of the regression line fitted to the frequency–speed and the firing rate-speed data points.

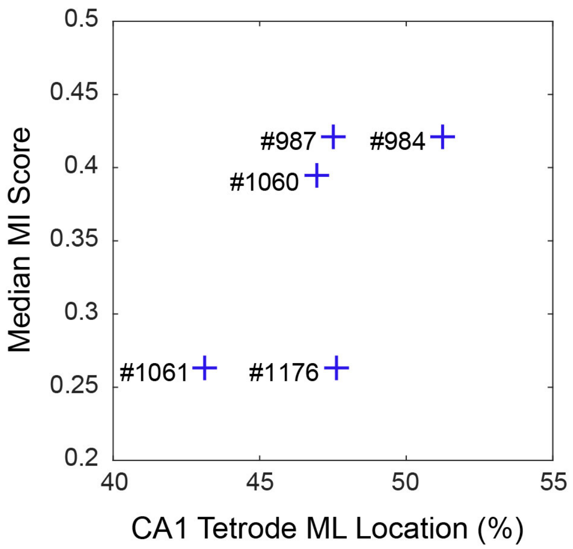

Supplementary Figure 12. Estimated CA1 tetrode locations and motor influence scores. Showing CA1 implants' medio-lateral (ML) locations (proportional to ML maximum extent of hippocampus) and the median motor influence (MI) scores of place cells recorded from those locations. (Histological estimation of location failed in animal 1015). More lateral locations are more proximal to CA3. The correlation coefficient between ML location and MI score did not reach significance (r = 0.65, p = 0.24).

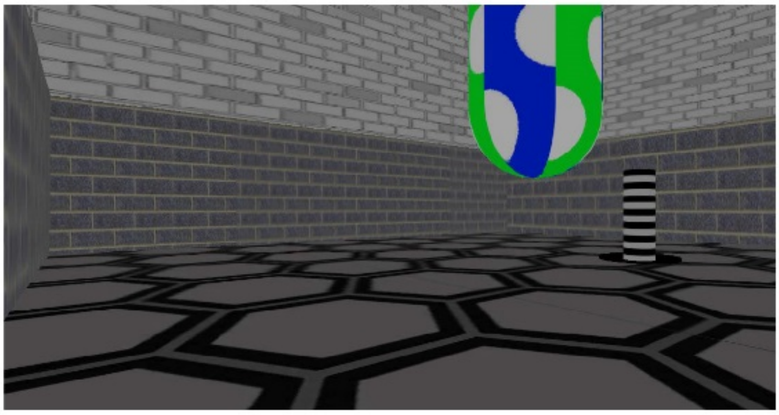

Supplementary Figure 13. Another view of the VR environment, opposite to that in Figure 1.

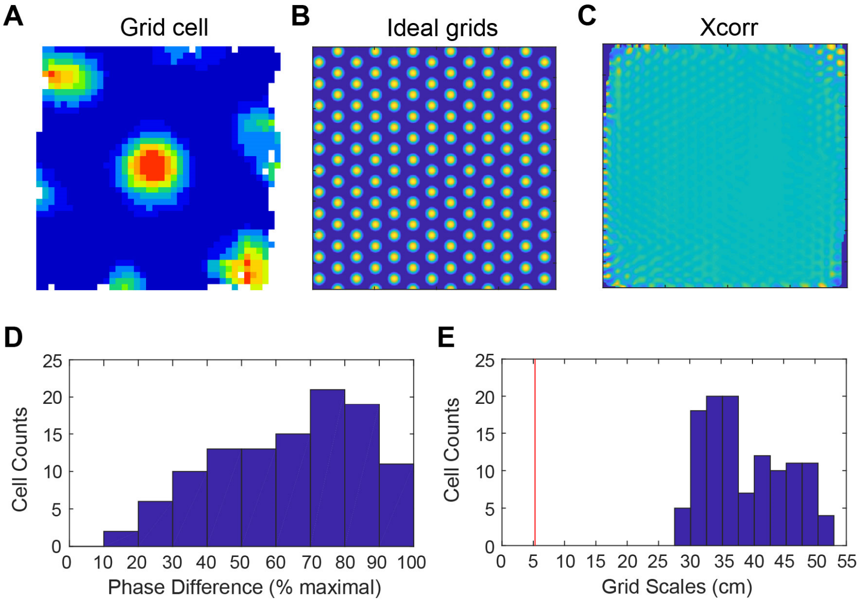

Supplementary Figure 14. Comparison of grid firing patterns with hexagonal virtual floor tiling pattern. (A) A rate map of an example grid cell. (B) A rate map of an ideal grid cell whose pattern matched the floor pattern. (C) Cross-correlation of the rate maps between (A) and (B). (D) The distribution of phase differences. (E) The distribution of grid scales. The red line indicates the grid scale of the floor pattern.
