# Supplementary material for: Differential influences of environment and self-motion on place and grid cell firing
Source: Nat Commun. 2019 Feb 7;10:630. doi: 10.1038/s41467-019-08550-1 (PMC6367320; doi:10.1038/s41467-019-08550-1)
Supplement: Supplementary file 3 — Description of Additional Supplementary Files [file 41467_2019_8550_MOESM3_ESM.docx]

Description of Additional Supplementary Files

**Supplementary Video 1:** Example of a mouse performing the random foraging task during recording of place and grid cells. Black and white striped poles mark the (randomly scattered) reward locations, a click can be heard when the valve delivers a drop of milk through the white tube, the LEDs are tracked by an overhead camera, the wires go to the electrophysiological recording system.
